# Histopathological Patterns of Lung Cancer in Iran: A Single-Center Study

**DOI:** 10.34172/aim.31133

**Published:** 2024-09-01

**Authors:** Babak Salimi, Sharareh Seifi, Adnan Khosravi, Sara Shiari, Raana Moradi, Babak Daneshfard, Maryam Mabani

**Affiliations:** ^1^Research Center of Thoracic Oncology (RCTO), National Research Institute of Tuberculosis and Lung Diseases (NRITLD), Shahid Beheshti University of Medical Science, Tehran, Iran; ^2^Chronic Respiratory Diseases Research Center, National Research Institute of Tuberculosis and Lung Diseases (NRITLD), Shahid Beheshti University of Medical Sciences, Tehran, Iran; ^3^Persian Medicine Network (PMN), Universal Scientific Education and Research Network (USERN), Tehran, Iran; ^4^Canadian College of Integrative Medicine, Montreal, Quebec, Canada

**Keywords:** Epidemiology, Iran, Lung cancer, Risk factor

## Abstract

**Background:**

Lung cancer (LC) is one of the leading causes of cancer-related deaths worldwide. In Iran, it is the second most common cause of cancer-related deaths for men and the third most common for women. This study aimed to examine the clinicopathological characteristics of Iranian patients with LC.

**Methods:**

Clinicopathological data of 1382 patients with primary LC diagnosed over 11 years (2012‒2023) at the "National Institute of Tuberculosis and Lung Disease" (NRITLD), Tehran, Iran, were retrospectively reviewed.

**Results:**

Adenocarcinoma was the most common type of cancer found in the patients (42.44%). The median age was 59.69 years (mean: 60.41 years) ranging 24–88 years. The mean male-to-female ratio was 3.65. Additionally, 65.84% of patients were smokers. The majority of patients (82.69 %) were diagnosed at an advanced stage (stage IV) of cancer.

**Conclusion:**

Although some of our findings are consistent with those of previous LC studies, there are some discrepancies, especially concerning the smoking status and median age of the Iranian patients. Therefore, additional clinical and epidemiological studies are needed to determine the impact of non-smoking factors, such as environmental exposure and genetic predisposition, on the development of LC.

## Introduction

 Lung cancer (LC) is the second most common type of cancer in both males and females and the leading cause of cancer-related deaths globally. Despite advances in treatment, the five-year survival rate remains at 19%, with 13% of all cancer cases and 24% of all cancer deaths attributable to LC.^1^ According to Garcia *et al.* (2007), the five-year survival rate for LC patients is only 15.9%.^2^ LC incidence is heavily influenced by geographic location, with higher rates observed in developed countries compared to underdeveloped ones. Moreover, the incidence of LC is on the rise in Asian countries.^3^ In Iran, LC is one of the most prevalent types of cancer, exhibiting an increasing trend and causing significant economic burdens. Its pattern varies across different geographical areas within Iran.^4^

 LC morbidity and mortality are more prevalent among men.^5^ This is likely due to several interconnected risk factors including smoking, diet, occupational exposures, environmental risks, familial history, and gender. Among these, smoking stands out as the most significant risk factor for LC.^6^ Studies have demonstrated that changing trends in smoking,^7^ diet,^8^ and other environmental and lifestyle factors,^9^ including air pollution,^10,11^ and occupational exposures,^10^ significantly influence the incidence and mortality of LC in Iran. These variations are further observed within different gender and histopathological subgroups. Our study emphasizes the critical need for accurate and timely data on these dynamic risk factors to improve diagnosis, treatment strategies, and patient outcomes in the Iranian context.

 This study investigates the epidemiological, pathological, and clinical attributes of 1832 primary LC cases in Iranian patients, who were diagnosed and treated at our specialized oncology center over a span of 11 years. Our objective is to uncover distinct patterns and features within this unique population, with the goal of enriching our knowledge of LC in Iran and subsequently informing more effective diagnosis and treatment strategies.

## Materials and Methods

 A retrospective, hospital-based cross-sectional study was conducted using data from 1,382 patients with primary LC diagnosed between August 2012 and June 2023 at the National Research Institute of Tuberculosis and Lung Disease (NRITLD). Patient diagnoses were confirmed by pathology specimens. NRITLD is an academic hospital affiliated with Shahid Beheshti University of Medical Sciences in Tehran, Iran. For this study, metastatic lung neoplasms originating from primary sites other than the lungs were excluded. Information on the patients’ demographic characteristics, smoking history, histological subtype, and cancer stage was obtained through chart reviews. Tissue specimens were categorized according to the 1981 World Health Organization (WHO) classification system for LC.^12^

 Non-small-cell lung cancer, not otherwise specified (NSCLC-NOS), is a diagnosis given to patients whose cancer cells do not clearly fall under the categories of adenocarcinoma or squamous cell carcinoma (SCC). Adenocarcinoma, large cell carcinoma, small cell lung carcinoma (SCLC), and SCC are the four forms of primary LCs. For patients with SCLC, a different staging system was used. Limited disease is defined as cancer confined to the same side of the chest as the tumor and can be covered within a single radiotherapy port. This corresponds to TNM stages I–IIIB. On the other hand, extensive disease is defined as cancer that has spread beyond the ipsilateral hemithorax (the side of the chest where the tumor is located) and metastasized to other parts of the body.^13^ For this study, a non-smoker was defined as someone who had smoked fewer than 100 cigarettes in their lifetime.^14^ However, exposure to passive smoking was not evaluated.

 A chi-square test was employed to assess the relationship between LC types and various factors, including age, gender, smoking status, and addiction. This analysis aimed to identify any statistically significant associations between these variables and the specific types of LC.

 Furthermore, an analysis of variance (ANOVA) was conducted to compare the average age of patients diagnosed with different LC types. This test helped determine whether the observed differences in average age were statistically significant or simply due to chance.

 A significance level of 5% was established for all statistical tests, and the analyses were conducted using SPSS version 27.

## Results

 The study included 1382 patients, of whom 1085 (78.45%, 60.41 ± 10.55) were men and 297 (21.47%, 57.06 ± 12.30) were women (male/female ratio: 3.65). The average age of the patients was 59.69 ± 11.03 years (60.41 years for men 57 years for women). The main characteristics of the patients are summarized in Table 1.

**Table 1:** Prevalence of Common Morphologies of Lung Cancers in Patients Referred to Masih Deneshvari Hospital between 2012-2022

	**All (n=1382)** **(21-88 y)**	**Male (n=1085)** **(24-88 y)**	**Female (n=297)** **(21-87 y)**	***P*** ** Value**
Age of diagnosis (Mean ± SD)	59.69 ± 11.03	60.41 ± 10.55	57.06 ± 12.30	0.0001
Histology				
Adenosquamous cell carcinoma	2 (.14%)	2 (100%)	0 (0%)	0.0001
Not otherwise specified	30 (2.16%)	25 (83.3%)	5 (16.7%)	
Primary Adenocarcinoma	587 (42.44%)	403 (68.7%)	184 (31.3%)	
Small cell carcinoma	319 (23.06%)	275 (86.2%)	44 (13.8%)	
Squamous cell carcinoma	444 (32.10%)	380 (85.6%)	64 (14.4%)	
Total	1382 (100)	1085 (100)	297 (100)	
Disease stage in non-small cell carcinoma				
I A	1 (.09 %)	1 (.12 %)	0 (0 %)	0.002
I B	2 (.18%)	1 (.12 %)	1 (.39 %)	
II A	29 (2.72 %)	23 (2.84 %)	6 (2.35 %)	
II B	5 (.47%)	4 (.49 %)	1 (.39 %)	
III A	75 (7.05 %)	63 (7.79 %)	12 (4.70%)	
III B	68 (6.39 %)	51 (6.31%)	17 (6.66%)	
III C	4 (.37 %)	4 (.49%)	0 (0 %)	
IV A	879 (82.69 %)	661 (81.80 %)	218 (85.49%)	
Total	1063 (100%)	808 (100%)	255 (100%)	
Disease stage in small cell carcinoma				
Limited stage	78 (24.45 %)	72 (26.08 %)	6 (13.95 %)	.058
Extensive stage	241 (75.54%)	204 (73.91%)	37 (86.04 %)	
Total	319(100%)	276 (100 %)	43 (100%)	
Smoking status				
Smoker	910 (65.84%)	857 (94.17 %)	53 (5.82 %)	0.0001
Non-smoker	472 (34.15%)	228 (48.30 %)	244(51.69 %)	
Addicted status				
Addicted	859 (62.15 %)	591 (54.52%)	28 (8.53 %)	0.0001
Not addicted	521 (37.69 %)	493 (45.47 %)	300 (91.46 %)	

**Figure 1:**
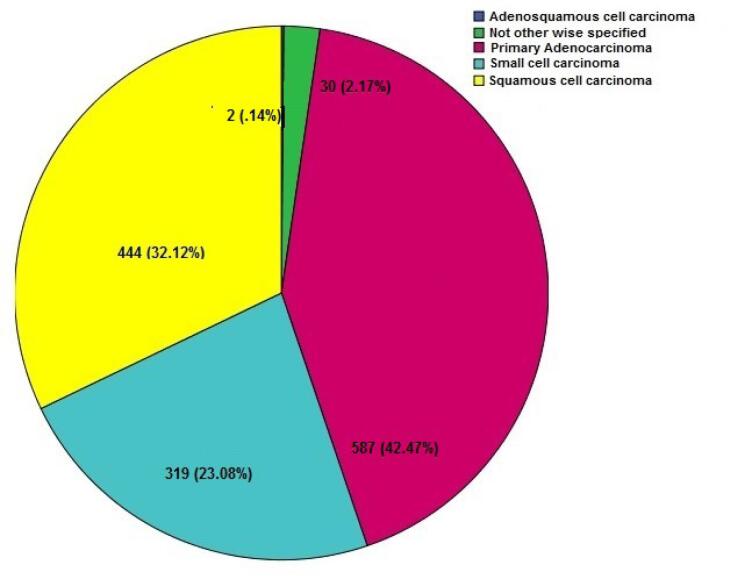
Histopathology Subtypes (n = 1382)

**Figure 2:**
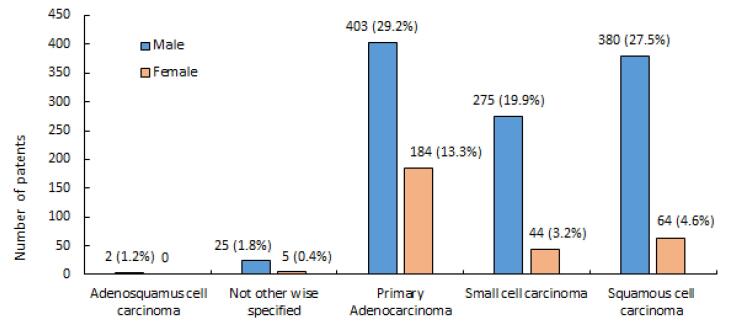
Distribution According to Gender and Histological Subtypes

 The vast majority (76.84%) of LC cases belonged to the NSCLC category (60.03 ± 11.26), far exceeding the prevalence of SCLC at 23.06% (58.56 ± 10.16). This skewed distribution shown in Figure 1 has significant implications for LC diagnosis, treatment, and prognosis. Adenocarcinoma was the most common pathology in LC patients of both genders (Table 1). Upon closer inspection, there were notable distinctions in the diagnoses based on gender (Figure 2). SCC was the most common kind of presentation in men, whereas SCLC was the most common type in women (Figure 2). This disparity highlights the possibility that different underlying biological factors and treatment considerations exist for different genders. Age, histology, and NSCLC stage at diagnosis were shown to differ significantly between men and women (*P* = 0.001, 0.0001, and 0.002, respectively). Approximately, 62.15% of the patients were drug abusers, and 65.84% were smokers. Smoking and addiction were significantly more prevalent among men than women (*P* = 0.0001).

 The analysis presented in Table 2 emphasizes that age plays a vital role in distinguishing the various subtypes of LC. Notably, patients with SCC exhibited a significantly higher average age compared to those with NSCLC-NOS (*P* = 0.038), primary adenocarcinoma (*P* = 0.0001), and small cell carcinoma (*P* = 0.0001). Further exploration is necessary to examine the biological and clinical consequences of age differences in LC diagnoses.

**Table 2:** Pairwise Comparison of Average Age in the Studied Groups

**Cancer Type**	**Cancer Type**	***P*** ** Value**
Adenosquamous cell carcinoma	Not otherwise specified	0.549
	Primary adenocarcinoma	0.669
	Small cell carcinoma	0.696
	Squamous cell carcinoma	0.924
Not otherwise specified	Adenosquamous cell carcinoma	0.549
	Primary adenocarcinoma	0.910
	Small cell carcinoma	0.855
	Squamous cell carcinoma	0.038
Primary Adenocarcinoma	Adenosquamous cell carcinoma	0.669
	Not otherwise specified	0.910
	Small cell carcinoma	0.994
	Squamous cell carcinoma	0.000
Small cell carcinoma	Adenosquamous cell carcinoma	0.696
	Not otherwise specified	0.855
	Primary adenocarcinoma	0.994
	Squamous cell carcinoma	0.000
Squamous cell carcinoma	Adenosquamous cell carcinoma	0.924
	Not otherwise specified	0.038
	Primary adenocarcinoma	0.000
	Small cell carcinoma	0.000

Table 3 shows the relative risk or odds ratio, along with 95% confidence intervals, for the relationship between smoking, addiction, age, and different types of LC. In the case of smoking, the incidence of not otherwise specified types of cancer was higher than that of the other types. In addition, the ratio of primary adenocarcinomas in smokers was 4.9 times higher than that of small cell carcinomas and 2.6 times higher than that of SCCs. The proportion of SCC was 1.9 times higher than that of small-cell carcinoma.

**Table 3:** Odds Ratio with a 95% Confidence Interval, Relating to the relationship Between Age, Smoking, Addiction, and Types of Lung Cancer

**Cancer Type**	**Cancer Type**	**OR ** **Age**	**OR ** **Addicted**	**OR Smoke**
Adenosquamous cell carcinoma	Not otherwise specified	1.161	-	-
	Primary adenocarcinoma	1.130	-	-
	Small cell carcinoma	1.138	-	-
	Squamous cell carcinoma	1.080	-	-
Not otherwise specified	Adenosquamous cell carcinoma	0.861	-	-
	Primary adenocarcinoma	0.982	0.792	1.324*
	Small cell carcinoma	0.980	1.320	6.133*
	Squamous cell carcinoma	0.953*	0.763	2.698*
Primary Adenocarcinoma	Adenosquamous cell carcinoma	0.885	-	-
	Not otherwise specified	1.018	1.263	0.755*
	Small cell carcinoma	1.002	1.324	4.978*
	Squamous cell carcinoma	0.970*	0.869	2.690*
Small cell carcinoma	Adenosquamous cell carcinoma	0.878	-	-
	Not otherwise specified	1.021	0.758	0.163*
	Primary adenocarcinoma	0.998	0.755	0.201*
	Squamous cell carcinoma	0.960*	0.687*	0.501*
Squamous cell carcinoma	Adenosquamous cell carcinoma	0.926	-	-
	Not otherwise specified	1.049*	1.310*	0.371*
	Primary Adenocarcinoma	1.031*	1.151	0.372*
	Small cell carcinoma	1.042*	1.456*	1.996*

* The mean difference is significant at the 0.05 level.

 In terms of age, the proportion of squamous cell cancers was higher than that of other types of LCs.

 In addition, the proportion of drug-related SCC was 1.3 and 1.4 times higher than those classified as “not otherwise specified” and small cell carcinoma, respectively.

## Discussion

 This study examined the demographic and clinicopathological characteristics of a cohort of Iranian LC patients. It aims to address the lack of comprehensive studies on LC in Iran that involve a large number of patients from a single institution. By utilizing data from 1382 patients, the study provides valuable insight into the regional trends and difficulties associated with this disease, which enhances our knowledge of the condition in this specific context. Our study reveals a notable difference in the diagnosis of LC between genders. Despite the average age of 59.69 years for the group, men have a significantly higher median age of 60.41 years as compared to women with an average age of 57 years (*P* < 0.05).

 It is important to note that adenocarcinoma was the most common type of LC, with males being affected 3.65 times more than females. Our research also reveals a significant difference in smoking habits, with men having a much higher prevalence of smoking at 94% compared to women at only 5%. Additionally, a staggering 76.84% of the patients in our study were diagnosed with advanced-stage LC.

 Refining early detection strategies and implementing gender-specific approaches are urgent to address the critical risk factor of age in cancer incidence. LC incidence is low before the age of 50 and then increases noticeably.^14,15^ This trend reflects the impact of aging populations in developed countries, where cancer disproportionately affects older people.^16^ Our study found that the average age of diagnosis for participants was 59.69 years, consistent with previous studies in Iran,^17-19^ Turkey (60 years),^20^ and India (56 years).^21^ It is worth noting that the age at which a diagnosis is made can vary based on geographical location. For instance, in Arab countries like Saudi Arabia, Kuwait, and the UAE, the average age at diagnosis is higher, at around 66 years.^22^ Similarly, developed countries like Canada (75 years),^23^ the USA (74 years),^24^ Japan (66.3 years),^25^ and Australia (71 years) also show a higher average age at diagnosis.^26^ The differences in racial/ethnic compositions may explain the observed variations in the mean age at diagnosis. Research conducted by Hamid et al^27^ suggests that these racial/ethnic characteristics might play a mediating role through genetic and lifestyle interactions. Nonetheless, it is crucial to consider other potential explanations, as well.

 It is possible that the differences in the average age of diagnosis of LC in Iran could be due to the high number of young smokers who died from cardiovascular diseases in the past. The improvements in cardiovascular care may have increased the survival rates of these individuals, which could have led to more cases of LC being diagnosed at a later stage in life. This could explain the observed increase in both incidence and median age at diagnosis in developing countries like Iran.^27^

 In our study, we found that women were diagnosed with LC at a younger age than men, which is consistent with previous research.^14,28-31^ Interestingly, despite being more likely to be lifetime non-smokers with shorter smoking histories and smoking fewer cigarettes per day, this disparity remained consistent among women in our study.^13,32,33^ Recent studies suggest that there may be other risk factors apart from smoking that contribute to the development of LC in women. Previous investigations have suggested that women’s genetic profile may increase their vulnerability to the carcinogenic effects of cigarette smoke and environmental toxins.^34^

 Significant disparities exist in the male/female ratio across different countries. Our study found a ratio of 3.65, consistent with prior researches in Iran (with a range of 2.79 to 5.09),^35^ Kuwait,^36^ and Japan.^37^ Certain countries, like Spain (which reported a ratio of 8.1)^38^ and India,^39^ have reported higher male/female ratios than others. Discrepancies in these ratios may be influenced by various factors, including smoking status, DNA repair capabilities, histological subtypes, and alcohol consumption.^40^

 There is a possibility that the disparity in LC incidence between men and women may be traced back to historical differences in smoking habits.^5^ Studies indicate that smoking is a major contributor to LC, responsible for 80%‒91% of cases in men and 45%‒69% of cases in women.^41,42^

 Even though smoking is the primary reason for LC, it is estimated that approximately a quarter of LC patients across the globe have never smoked.^14^ Non-smoking LC patients are mostly women with adenocarcinoma and a younger age of onset.^43^ Notably, the smoking prevalence in Iran fluctuates between 23.9% and 26% in men and 1.7% and 3.6% in women.^44,45^

 In Iran, social stigma associated with smoking in women might be the reason why only 5.82% of the 297 females were smokers in a recent study. Our study highlights the fact that LC is influenced by a combination of genetic and environmental factors. Another study found that LC in Asian women who have never smoked is associated with variations in three genomic locations on chromosomes 6 and 10. Asian women may be more vulnerable to the effects of second-hand smoke.^46^

 Certain genes, such as GRPR, are expressed more frequently in non-smoking women than in men and have been linked to bronchial cell proliferation.^47^ Similarly, polymorphisms in the ER gene have been connected to lung adenocarcinoma in women who have never smoked. These observations suggest that genetic factors may play a role in the development of LC, regardless of smoking habits.^48^

 In recent years, the incidence of adenocarcinoma has surpassed that of SCC.^49^ This may be due to changes in cigarette composition and smoking method.^50^ Technological advancements have shifted cases from large cell carcinoma to adenocarcinoma due to improvements in identifying peripheral pulmonary lesions, modifications in the WHO classification, and enhancements in staining mucin-producing cells.^51^ Another factors that might contribute to the increase in adenocarcinoma cases is air pollution, particularly nitrogen oxides.^52^

 The most frequently observed pathology was adenocarcinoma, which is consistent with previous research.^17,53,54^ In our study, the majority of patients were at advanced stages, which is consistent with international reports.^55^

 While this retrospective study offers valuable insight, it is important to recognize its limitations. Firstly, the accuracy of the smoking data might be compromised due to its retrospective nature. Secondly, we were unable to assess the impact of other potential contributing factors, such as passive smoking, air pollution, or substances like silica or asbestos. Moreover, studies suggest that individuals with a family history of LC diagnosed before the age of 60 are at a higher risk of developing the disease.^56^ However, it was not possible to investigate the family history of the patients in this study as the available data was incomplete.

 There are differing opinions regarding the role of infection as a causative agent for LC. Some researchers believe that certain viruses, such as human papillomavirus,^57^ Epstein-Barr virus,^58^ human cytomegalovirus, simian virus 40 (SV40), and measles virus, are associated with LC.^9^ Pulmonary tuberculosis, a common infection in Iran, has also been associated with LC.^59^ It is important to conduct further studies to determine the association between different infections and LC across various geographic regions. This study was carried out on patients who were referred to the tertiary center of NRTLD from all over Iran. However, it is important to note that the findings may not precisely represent the clinicopathologic and demographic characteristics of all Iranian patients diagnosed with LC.

## Conclusion

 The clinicopathological features of Iranian LC patients are highlighted in this research, revealing potential differences in patterns and triggers between developing nations and developed countries. Our research is in line with studies carried out in regional or developing countries. It is worth mentioning that our study discovered that patients diagnosed with LC in Iran were younger and had a lower rate of smoking compared to other studies. This variation could be due to other factors, such as genetic predisposition, endemic infectious diseases, and environmental exposures. In order to gain a better understanding of the link between smoking and LC in developing countries, it is essential to establish a thorough national cancer registry. This would enable the sharing of data and resources, promote research, and aid the development of effective strategies to combat LC, through regional and international cooperation.
